# Effectiveness of a Bariatric-Specific Multivitamin Versus Conventional Targeted Supplementation for Preoperative Micronutrient Deficiency Correction in Bariatric Surgery Candidates: A Multicenter Retrospective Cohort Study

**DOI:** 10.3390/nu18071047

**Published:** 2026-03-25

**Authors:** Luigi Schiavo, Monica Mingo, Gianluca Rossetti, Farnaz Rahimi, Simona Bo, Luigi Cobellis, Francesco Cobellis, Emmanuele Giglio, Lilia Bertolani, Vincenzo Pilone

**Affiliations:** 1 Department of Medicine, Surgery and Dentistry “Scuola Medica Salernitana”, University of Salerno, 84081 Baronissi, Italy; mmingo@unisa.it; 2 General and Bariatric Surgery Unit, Abano Terme Policlinic, 35031 Padova, Italy; gianlucarossetti@yahoo.it; 3 Dietetic Unit, Città della Salute e della Scienza Hospital, 10126 Turin, Italy; frahimi@cittadellasalute.to.it; 4 Department of Medical Sciences, University of Torino, 10124 Turin, Italy; simona.bo@unito.it; 5 Unit of General Surgery, Casa Di Cura “Prof. Dott. Luigi Cobellis”, 84078 Vallo Della Lucania, Italy; luicobellis@yahoo.it (L.C.); cobellisfc@gmail.com (F.C.); 6 Department of Bariatric Surgery, Clinical Institute “Beato Matteo”, 27029 Vigevano, Italy; giglio.emmanuele1@gmail.com (E.G.); lilia.bertolani@yahoo.it (L.B.); 7 Public Health Department, University of Naples Federico II, 80131 Naples, Italy; vincenzo.pilone@unina.it

**Keywords:** micronutrient deficiencies, bariatric surgery, obesity, preoperative nutrition, dietary supplements

## Abstract

Background: Micronutrient deficiencies (MD) are highly prevalent among candidates for bariatric surgery (BS) and are associated with adverse perioperative and postoperative outcomes. Although guidelines recommend systematic preoperative screening and correction, conventional targeted supplementation (CTS) often requires multiple products, potentially limiting adherence and delaying surgical readiness. Bariatric-specific multivitamins (BSM) may simplify nutritional management, but their real-world effectiveness for preoperative correction of multiple MD remains insufficiently investigated. Objective: To compare the effectiveness, efficiency, and adherence of a BSM versus CTS for preoperative correction of multiple MD in BS candidates. Methods: This retrospective multicenter cohort study included 1560 adults with obesity evaluated for BS between 2020 and 2024 across three Italian bariatric centers. The primary efficacy analysis was restricted to patients presenting with ≥3 laboratory-confirmed MD at baseline. Patients treated between 2020 and 2022 received individualized CTS using multiple products, whereas those treated between 2023 and 2024 received a single BSM. Biochemical follow-up was scheduled at 4 and 8 weeks. The primary outcome was the achievement of complete biochemical correction of all baseline deficiencies at the predefined 4-week follow-up assessment (composite endpoint). Secondary outcomes included supplementation burden and self-reported adherence. Early correction rates were compared using absolute risk differences and risk ratios; adjusted associations were evaluated using multivariable regression models including center and baseline deficiency burden. As a supplementary analysis, the patient-level proportion of baseline deficiencies corrected at 4 weeks was also evaluated. Results: Among patients with ≥3 baseline deficiencies (*n* = 216), complete biochemical correction at 4 weeks was achieved in 55/134 patients (41.0%) in the BSM group and in 13/82 patients (15.9%) in the CTS group, corresponding to an absolute risk difference of 25.2 percentage points (95% CI 7.8–40.0) and a risk ratio of 2.59 (95% CI 1.51–4.44). In adjusted analyses accounting for center and baseline deficiency pattern, BSM use remained independently associated with early complete correction (adjusted absolute risk difference 26.3 percentage points; adjusted risk ratio 2.69). Sensitivity analyses restricting follow-up timing and excluding early calendar periods yielded consistent results. The mean proportion of baseline deficiencies corrected per patient at 4 weeks was higher in the BSM group compared with CTS (0.74 ± 0.25 vs. 0.54 ± 0.30). Compared with CTS, BSM was associated with lower supplementation burden (1 vs. 3.5 supplements on average) and higher adherence (92% vs. 70%). Conclusions: In a real-world multicenter cohort of BS candidates with ≥3 baseline MD, a simplified preoperative supplementation strategy based on a BSM was associated with a significantly higher probability of complete biochemical correction at 4 weeks, lower supplementation burden, and higher reported adherence compared with CTS. Although complete correction was not universal at 4 weeks, BSM significantly increased the likelihood of achieving early multi-deficiency normalization. Given the non-concurrent observational design, these findings should be interpreted as hypothesis-generating and warrant confirmation in prospective studies with concurrent cohorts.

## 1. Introduction

Bariatric surgery (BS) represents the most effective long-term therapeutic option for the treatment of severe obesity and its associated metabolic comorbidities [1,2,3]. However, despite its well-established benefits on weight loss, glycemic control, and cardiovascular risk, BS is consistently associated with a high burden of micronutrient deficiencies (MD) [4], which may already be present before surgery [5,6,7] and can be further exacerbated in the postoperative period due to procedure-related anatomical and functional changes that impair nutrient absorption [4]. Paradoxically, individuals with obesity frequently present with multiple MD despite excessive caloric intake [8,9]. Previous studies have demonstrated the high preoperative prevalence of deficiencies in vitamin D, iron, folate, vitamin B12, zinc, and selenium among candidates for BS, reflecting poor dietary quality, chronic low-grade inflammation, metabolic dysregulation, and altered nutrient distribution related to adipose tissue sequestration [8,9]. Importantly, these deficiencies rarely occur in isolation and frequently coexist within the same individual, contributing to a complex state of preoperative nutritional vulnerability [10].

In a prospective preoperative screening study, up to 80% of bariatric candidates were found to have at least one MD, and a substantial proportion exhibited multiple concurrent deficiencies [8]. Patients presenting with multiple baseline deficiencies may represent a particularly vulnerable subgroup requiring structured preoperative optimization. Growing evidence indicates that inadequate correction of MD before surgery represents a major determinant of early postoperative nutritional abnormalities and related complications [6,7]. Preoperative deficiencies have been associated with anemia, impaired wound healing, fatigue, neurological manifestations, and delayed postoperative recovery, potentially increasing early postoperative morbidity [11]. Moreover, bariatric procedures, through reduced gastric acid secretion, altered gastrointestinal anatomy, and decreased absorptive capacity, may exacerbate pre-existing deficiencies or precipitate new ones in the immediate postoperative phase [12]. Previous clinical observations have shown that patients who undergo systematic correction of MD before surgery are less likely to develop early postoperative deficiencies, even when standardized postoperative supplementation is prescribed, highlighting the critical role of preoperative nutritional status in shaping postoperative outcomes [9].

From a practical perspective, both prospective and retrospective studies have demonstrated that correction of multiple MD can be achieved within a relatively short preoperative timeframe, independently of dietary modifications or concomitant pharmacological treatments [6,8]. These findings support the feasibility of systematic preoperative nutritional optimization within routine clinical pathways and reinforce the concept that preoperative correction should be considered an integral component of surgical preparation rather than an optional intervention [7]. Despite this evidence, current clinical practice remains highly heterogeneous. While international guidelines recommend routine screening and correction of MD before BS, they provide limited guidance regarding the optimal supplementation strategy, timing, and formulation [10]. In particular, whether a simplified bariatric-specific multivitamin (BSM) approach may be as effective as, or superior to, conventional targeted supplementation (CTS) protocols in the preoperative setting remains largely unexplored.

Moreover, real-world implementation of preoperative correction is frequently hindered by regimen complexity and poor adherence, which represent major and often underestimated barriers to effective nutritional optimization [13]. Treatment adherence, in particular, is a well-recognized challenge in bariatric populations [4]. Complex supplementation regimens requiring multiple products may reduce compliance, delay correction, and increase the risk of incomplete nutritional optimization before surgery. Simplified supplementation strategies may therefore offer not only biochemical benefits but also meaningful improvements in adherence and preoperative pathway efficiency. Recent narrative reviews have emphasized that, despite clear recommendations for preoperative micronutrient screening and correction, real-world implementation remains inconsistent, largely due to regimen complexity and suboptimal adherence [7].

Against this background, the present study aimed to compare the effectiveness of a bariatric-specific multivitamin (BSM) versus conventional targeted supplementation (CTS) for the preoperative correction of multiple MD in a large real-world cohort of BS candidates. Specifically, the primary analysis focused on patients presenting with ≥3 laboratory-confirmed baseline deficiencies, evaluating complete correction of all baseline deficiencies at 4 weeks. Secondary objectives included assessment of supplementation burden, treatment adherence, and predictors of early micronutrient normalization. By addressing this clinically relevant and under-investigated gap, the study aims to inform the optimization of preoperative nutritional management and improve surgical readiness within bariatric care pathways.

## 2. Materials and Methods

### 2.1. Study Design and Data Source

**Figure 1:**
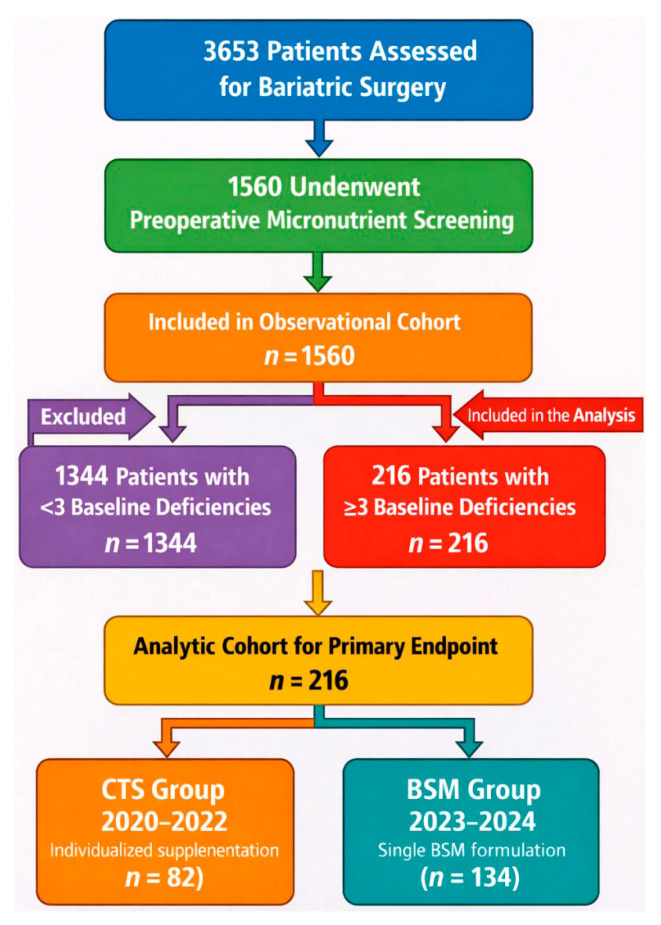
STROBE-informed flow diagram illustrating patient screening, eligibility assessment, and cohort selection across three Italian bariatric centers between January 2020 and December 2024. CTS, conventional targeted supplementation; BSM, bariatric-specific multivitamin.

This retrospective multicenter cohort study included adult patients with obesity who were evaluated for BS between January 2020 and December 2024 across three high-volume Italian bariatric centers (Salerno, Turin, and Vigevano), all accredited as Centers of Excellence by the Italian Society of Bariatric and Metabolic Surgery and Multidisciplinary Obesity Therapies (SICOB). The participating centers were selected because they followed identical preoperative screening protocols and laboratory cut-offs. During the study period, all patients underwent standardized preoperative nutritional and biochemical screening as part of routine clinical care. Patients were eligible for BS if they met the following criteria: age between 18 and 65 years; body mass index (BMI) ≥ 40 kg/m^2^, or BMI ≥ 35 kg/m^2^ with at least one obesity-related comorbidity [1,3,14]. For the primary efficacy analysis, patients were eligible only if they presented with at least three concurrent laboratory-confirmed MD at baseline. The threshold of ≥3 concurrent MD was selected to identify a clinically relevant subgroup of patients with a higher baseline nutritional burden. In this population, conventional targeted supplementation typically requires multiple concurrent products, increasing regimen complexity and potentially affecting adherence. Focusing on this subgroup allowed us to evaluate the potential advantages of simplified supplementation strategies in a clinically meaningful high-risk population. Exclusion criteria were pregnancy, known chronic renal or hepatic failure, inflammatory bowel disease, active malignancy, and the use of medications known to interfere with micronutrient absorption or metabolism (including corticosteroids, diuretics, antiepileptics, and bisphosphonates). Of the 3653 patients evaluated for BS during the study period, 1560 underwent standardized preoperative screening and were included in the overall observational cohort. Among these, 216 patients met the predefined criterion of ≥3 baseline deficiencies and constituted the analytic cohort for the primary endpoint. A STROBE-informed study flow diagram summarizing patient screening, eligibility, and inclusion is provided in Figure 1. Based on the supplementation strategy routinely adopted during the corresponding period, patients were retrospectively assigned to one of two groups: the CTS group, including patients treated between 2020 and 2022 with individualized correction protocols using multiple supplements (analytic cohort *n* = 82), and the BSM group, including patients treated between 2023 and 2024 with a single BSM formulation (analytic cohort *n* = 134). Although the two supplementation strategies were implemented during different calendar periods, clinical pathways, preoperative screening protocols, laboratory assessment methods, and follow-up schedules remained unchanged throughout the study period, thereby reducing, although not completely eliminating, the potential impact of temporal bias. No modifications in clinical staffing, laboratory platforms, or diagnostic thresholds for MD occurred during the observation window. All patients were followed for a total of 8 weeks after initiation of supplementation. Follow-up biochemical assessments were routinely scheduled at fixed time points (4 and 8 weeks), in accordance with institutional clinical practice. The primary endpoint was evaluated at the predefined 4-week follow-up visit.

### 2.2. Anthropometric and Biochemical Assessment

Anthropometric measurements were obtained under standardized conditions by trained healthcare personnel. Body weight was measured in the fasting state, with patients wearing light clothing and no shoes, using a calibrated digital scale (Seca 869, Intermed, Milan, Italy). Height was measured using a wall-mounted stadiometer (Seca 206, Intermed, Milan, Italy). BMI was calculated as body weight (kg) divided by height squared (m^2^). Venous blood samples were collected after an overnight fast and analyzed in certified clinical laboratories according to standardized procedures. All biochemical analyses were performed using routine automated methods within accredited hospital laboratories. The same laboratory platforms, analytical methods, and diagnostic thresholds were maintained throughout the study period across all participating centers. For the purposes of the present comparative analysis, the standardized preoperative micronutrient panel used to define baseline deficiencies included 25-hydroxyvitamin D [25(OH)D], iron, folate, vitamin B12, and zinc.

These micronutrients were selected because they represent those most frequently reported as deficient in patients with obesity and in candidates for BS, and are routinely included in standardized preoperative nutritional screening protocols [7,8,10]. MD were defined according to established laboratory reference ranges and international clinical guidelines [4,10]. Deficiency status was operationalized as a binary variable (deficient vs. within reference range) for each micronutrient at baseline and at follow-up. For inclusion in the analytic cohort, patients were required to present with at least three concurrent laboratory-confirmed deficiencies among the five evaluated micronutrients at baseline. This approach ensured the selection of a homogeneous subgroup with clinically relevant and multifactorial nutritional impairment. At follow-up, correction of a baseline deficiency was defined as normalization of the corresponding laboratory parameter according to the same predefined reference thresholds. The primary endpoint, complete correction of baseline deficiencies, required normalization of all micronutrients that were deficient at baseline for each individual patient.

### 2.3. Micronutrient Correction Protocols

Patients in the CTS group received individualized supplementation based on the specific MD identified at baseline. Standard therapeutic dosages were prescribed as follows: vitamin B12, 500 µg/day; folate, 15 mg/day; iron, 105 mg/day; zinc, 30 mg/day; and vitamin D, 10,000 IU/day. Additional supplements were prescribed when clinically indicated. Treatment duration ranged from 4 to 8 weeks within the study observation window, depending on biochemical response assessed at the predefined follow-up visits. In the CTS group, supplementation was tailored to the pattern and severity of baseline deficiencies, often requiring multiple concomitant products. Patients in the BSM group received a single daily dose of a commercially available high-potency BSM formulation (classified as a Food for Special Medical Purposes [FSMP]) (Bariatrifast^®^, registered in the Italian national FSMP database, authorization code 935988156, produced by Bioitalia S.r.l., Rome, Italy) according to routine clinical practice [15]. The daily dose of the BSM provided vitamin D 175 µg (approximately 7000 IU), iron 65 mg, folic acid 400 µg, vitamin B12 500 µg, and zinc 10 mg. Although the absolute dosages differed between CTS and BSM strategies, both approaches provided micronutrient amounts within ranges recommended by international bariatric nutrition guidelines for correction of documented deficiencies [4,10]. The CTS strategy relied on higher single-nutrient doses for specific deficiencies, whereas the BSM strategy provided a combined formulation with moderate-to-high doses of multiple micronutrients in a single product. Biochemical reassessment was performed at the predefined 4- and 8-week follow-up visits. Supplementation was continued until normalization of all baseline-deficient parameters. For the primary endpoint, complete correction was defined as normalization—at the 4-week assessment—of all micronutrients that were deficient at baseline in each individual patient. Supplementation burden was operationalized as the mean number of distinct daily supplement products required per patient during the correction phase. Treatment adherence was assessed through structured self-report at follow-up visits and expressed as the proportion of patients reporting full daily intake of the prescribed regimen.

### 2.4. Outcome Measures

The primary outcome was achievement of complete biochemical correction of all baseline deficiencies at the predefined 4-week follow-up assessment (composite endpoint). For the primary efficacy analysis, the endpoint was specifically evaluated at the 4-week follow-up visit among patients with ≥3 baseline deficiencies. Given the fixed follow-up schedule, time to correction was defined categorically as the achievement of complete biochemical normalization at the 4-week or 8-week visit, rather than as a continuous time-to-event variable. Accordingly, the analysis focused on the proportion of patients achieving complete correction at each predefined visit, without formal survival modeling. Secondary outcomes included the number of supplements used per patient and treatment adherence. Supplementation burden was quantified as the mean number of distinct daily supplement products prescribed during the correction phase. Treatment adherence was recorded as the percentage of patients reporting full compliance with the prescribed regimen at follow-up, as documented in clinical records. Due to the retrospective design of the study, no validated adherence questionnaires were administered. This reliance on self-reported data may introduce recall or reporting bias, potentially overestimating true adherence rates. Nonetheless, adherence assessment procedures were identical across both groups, thereby limiting the likelihood of differential misclassification. In addition to crude comparisons, adjusted analyses were performed to account for potential confounding factors, including center and baseline deficiency burden, to evaluate the independent association between supplementation strategy and early complete correction.

### 2.5. Statistical Analysis

Continuous variables are presented as mean ± standard deviation (SD), while categorical variables are expressed as absolute frequencies and percentages. Between-group comparisons were performed using the independent Student’s *t*-test or the Mann–Whitney U test for continuous variables, as appropriate, and the chi-square test or Fisher’s exact test for categorical variables. Early correction rates at 4 weeks were additionally compared using absolute risk differences and risk ratios with corresponding 95% confidence intervals (CIs). A multivariable regression model was constructed to evaluate the independent association between supplementation strategy and early complete correction of MD at 4 weeks, adjusting for center and baseline deficiency burden. Adjusted absolute risk differences and risk ratios were estimated using generalized linear models with appropriate link functions. As a supplementary analysis, the patient-level proportion of baseline MD corrected at 4 weeks was calculated as the number of baseline deficiencies normalized at 4 weeks divided by the total number of baseline deficiencies per patient. Between-group comparisons of this proportional continuous outcome were performed using the Mann–Whitney U test, given the non-normal distribution of the variable. Effect size was additionally quantified using Cliff’s delta as a non-parametric measure of stochastic dominance. This supplementary outcome was intended to provide a more granular assessment of early multi-parameter biochemical response beyond the binary composite endpoint. Sensitivity analyses were conducted by restricting follow-up timing and excluding early calendar periods to assess the robustness of the findings. Statistical significance was set to *p* < 0.05. All analyses were performed using IBM SPSS Statistics version 26.0 (IBM Corp., Armonk, NY, USA). Given the retrospective observational design of the study, no formal a priori sample size calculation was performed. The sample analyzed was determined by the number of eligible patients available during the predefined study period.

### 2.6. Ethical Considerations

The study was conducted in accordance with the Declaration of Helsinki. Given the retrospective design and the analysis of anonymized data collected as part of routine clinical care, the study was classified as non-interventional. Formal ethical committee approval was waived in accordance with institutional regulations. All procedures were performed within standard clinical practice, and no additional interventions or laboratory assessments were introduced for research purposes. Written informed consent for clinical treatment and the use of anonymized data for research purposes was obtained from all participants at the time of surgical evaluation. Data were processed in compliance with applicable national regulations and the European General Data Protection Regulation (GDPR), ensuring confidentiality and data protection throughout the study.

## 3. Results

### 3.1. Baseline Characteristics

A total of 1560 patients underwent standardized preoperative screening during the study period and constituted the overall observational cohort, of whom 620 were assigned to the CTS group and 940 to the BSM group. For the primary efficacy analysis, 216 patients met the predefined criterion of ≥3 laboratory-confirmed MD at baseline and constituted the analytic cohort (CTS *n* = 82; BSM *n* = 134). Baseline demographic and clinical characteristics of the overall cohort are summarized in Table 1. The BSM group was slightly younger than the CTS group (43 ± 6 vs. 45 ± 13 years) and had a marginally higher mean body mass index (54.6 ± 11.4 vs. 52.3 ± 13.9 kg/m^2^). The proportion of female patients was similar between groups (67% in CTS vs. 70% in BSM). With respect to baseline MD in the overall cohort, vitamin D deficiency was the most prevalent condition in both groups, with a slightly higher prevalence in the BSM group (70% vs. 65%). Iron deficiency was observed in 50% of patients in the BSM group and 45% in the CTS group. The prevalence of folate, vitamin B12, and zinc deficiencies was comparable between groups. Within the analytic cohort of patients with ≥3 baseline deficiencies, the distribution of age, sex, BMI, and baseline deficiency patterns was broadly comparable between CTS and BSM groups (Table 2). Although minor differences in selected variables were observed, the overall demographic and biochemical profiles were clinically similar between groups. To account for potential residual confounding, center, baseline deficiency count, and individual baseline deficiency indicators were included in multivariable adjusted analyses. Age, sex, and BMI were additionally explored as covariates in sensitivity models. Overall, both cohorts represented clinically comparable populations of BS candidates with a high burden of concurrent MD.

### 3.2. Correction of MD

**Figure 2:**
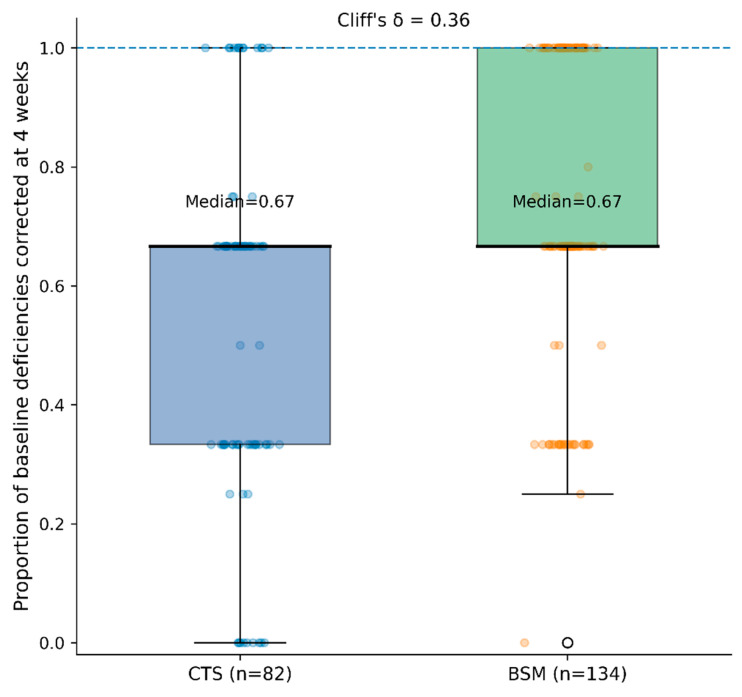
Patient-level proportion of baseline MD corrected at 4 weeks among patients presenting with ≥3 baseline deficiencies. The proportion was calculated as the number of baseline deficiencies normalized at 4 weeks divided by the total number of baseline deficiencies per patient. Boxes represent the interquartile range, horizontal lines indicate the median, and dots represent individual patients. The dashed horizontal line indicates complete correction (proportion = 1.0). Between-group differences were quantified using Cliff’s delta.

At the 4-week assessment, complete biochemical correction of all baseline MD was observed in 55/134 patients (41.0%) in the BSM group and in 13/82 patients (15.9%) in the CTS group (Table 3). Importantly, the primary endpoint required concurrent normalization of all baseline deficiencies. Because the primary analysis was restricted to patients with ≥3 laboratory-confirmed deficiencies at baseline, this composite definition represents a clinically stringent outcome measure. To provide a more granular understanding of early biochemical response, correction dynamics were further analyzed according to the number of deficiencies normalized per patient at 4 weeks (Table 3). In addition, the patient-level proportion of baseline deficiencies corrected at 4 weeks (number of corrected baseline deficiencies divided by the total number of baseline deficiencies per patient) was evaluated as a supplementary continuous measure of early response (Figure 2). The median proportion corrected was higher in the BSM group compared with CTS (median [IQR]: 0.67 [0.67–1.00] vs. 0.67 [0.33–0.67]), with a moderate effect size (Cliff’s δ = 0.36). The between-group difference was statistically significant (Mann–Whitney U test, *p* < 0.001), indicating a broader early multi-parameter improvement beyond the binary composite endpoint. In the CTS group, the majority of patients achieved partial biochemical improvement, with normalization of at least one baseline deficiency observed in most individuals; however, only 15.9% achieved complete multi-deficiency correction within 4 weeks. In contrast, patients receiving BSM demonstrated a significantly higher probability of achieving simultaneous normalization of all baseline-deficient parameters at the first scheduled follow-up, although complete correction was not universal. When individual micronutrients were examined separately, improvements in serum concentrations of vitamin D, iron, folate, vitamin B12, and zinc were observed in both groups, indicating that early biochemical response occurred under both supplementation strategies. Thus, the between-group difference observed at the 4-week assessment primarily reflects a higher likelihood of achieving full composite normalization in the BSM group, rather than absence of early therapeutic response in the CTS group. The absolute risk difference at 4 weeks was 25.2 percentage points (95% CI 7.8–40.0), corresponding to a risk ratio of 2.59 (95% CI 1.51–4.44). Adjusted analyses accounting for center and baseline deficiency burden yielded consistent findings, confirming the independent association between BSM use and early complete correction. By the 8-week assessment, rates of complete correction increased in both groups; however, complete normalization was not uniformly achieved in all patients within the analytic cohort, indicating that differences between strategies were primarily related to early correction efficiency rather than eventual therapeutic capacity.

### 3.3. Supplementation Burden and Adherence

Patients receiving CTS required a higher number of supplements to achieve complete correction compared with those treated with the BSM (mean ± SD: 3.5 ± 1.2 vs. 1.0 ± 0.0 supplements per patient, respectively; Table 4). Treatment adherence, assessed as part of routine clinical follow-up based on patient self-report documented in medical records, was higher in the BSM group compared with the CTS group (92% vs. 70%, respectively; Table 4). The absolute difference in adherence rates was 22 percentage points, favoring the BSM strategy. Overall, these findings indicate that the simplified supplementation strategy was associated with a markedly lower treatment burden and improved real-world adherence. The combination of reduced regimen complexity and higher adherence may partly explain the higher probability of early complete biochemical correction observed in the BSM group.

### 3.4. Factors Associated with Early MD Correction

At the 4-week assessment, the absolute risk difference for complete correction of MD between treatment groups was 25.2 percentage points (95% CI 7.8–40.0), indicating a substantially higher early correction rate in the BSM group compared with the CTS group. In multivariable analyses adjusted for center, baseline deficiency count, and individual baseline deficiency indicators, the use of a BSM emerged as the factor independently associated with early correction of MD within 4 weeks (Table 3). Adjusted risk differences were estimated using linear probability models with heteroskedasticity-robust standard errors, and adjusted risk ratios were obtained using modified Poisson regression with robust variance. Treatment with a BSM was associated with a markedly higher likelihood of achieving complete correction at the 4-week assessment (adjusted absolute risk difference 26.3 percentage points; adjusted risk ratio 2.69 [95% CI 1.58–4.57]). No evidence of model instability or separation was observed. No relevant independent associations were identified for age, sex, body mass index, or baseline number of MD in sensitivity models. Consistent findings were observed in sensitivity analyses using alternative modeling approaches, confirming the robustness of the association between supplementation strategy and early correction. To further explore potential center-level heterogeneity, center-stratified analyses were performed (Supplementary Table S1 and Supplementary Figure S1). The association between BSM use and early complete correction at 4 weeks was directionally consistent across all three participating centers. Although center-specific confidence intervals were wide due to limited subgroup sample sizes, no reversal of effect was observed, suggesting that the primary association was not driven by a single-site practice pattern. Absolute risk difference refers to complete micronutrient correction at the 4-week assessment. Given the composite and time-fixed nature of the outcome, estimates reflect differences in early correction efficiency within predefined follow-up visits rather than continuous time-to-event dynamics.

## 4. Discussion

The present study demonstrates that, in a large multicenter cohort of bariatric surgery (BS) candidates presenting with multiple MD, the use of a bariatric-specific multivitamin (BSM) was associated with a significantly higher probability of achieving complete biochemical correction at 4 weeks, a lower supplementation burden, and higher reported treatment adherence compared with conventional targeted supplementation (CTS). Importantly, these findings were observed under routine clinical care conditions rather than within a controlled experimental setting, thereby enhancing the real-world relevance and translational applicability of the results. In the analytic cohort of patients presenting with ≥3 baseline deficiencies, complete biochemical correction at 4 weeks was achieved in 41.0% of BSM-treated patients compared with 15.9% of those receiving CTS, corresponding to an absolute risk difference of 25.2 percentage points and a risk ratio of 2.59. Although complete normalization was not universal in either group at this early time point, correction rates increased progressively over the predefined 8-week follow-up period in both cohorts. Therefore, the observed differences should be interpreted primarily in terms of greater efficiency in early correction within routine clinical practice, rather than as evidence of intrinsic pharmacological superiority of one supplementation strategy over the other. Earlier correction of MD may have relevant clinical implications. Faster biochemical normalization may facilitate surgical readiness and streamline preoperative optimization pathways within bariatric programs. In practical terms, more rapid correction of deficiencies may allow multidisciplinary teams to complete the nutritional optimization phase more efficiently, potentially reducing delays in surgical scheduling related to unresolved deficiencies. Moreover, adequate micronutrient status prior to surgery may improve physiological resilience during the perioperative period, potentially reducing risks related to anemia, impaired wound healing, or metabolic instability. Although these outcomes were not directly evaluated in the present study, earlier correction of deficiencies could represent an important organizational and clinical advantage in bariatric care pathways. A key methodological consideration of the present study is the non-concurrent implementation of the two supplementation strategies, with CTS used between 2020 and 2022 and BSM introduced between 2023 and 2024. Although preoperative screening protocols, laboratory assays, diagnostic thresholds, follow-up schedules, and overall clinical care pathways remained unchanged throughout the study period, the temporal separation between cohorts introduces the possibility of secular trends influencing the results. For example, increased clinical awareness of MD over time, progressive optimization of multidisciplinary bariatric care pathways, or improvements in patient counseling and education may have contributed to higher correction rates during the later study period independently of the supplementation strategy itself. In practical terms, greater clinician awareness could lead to more consistent counseling regarding supplement use, closer monitoring of adherence, or earlier identification of patients requiring intensified nutritional support. Because supplementation strategy was structurally determined by calendar period, calendar time and treatment exposure were intrinsically collinear variables and could not be simultaneously included in multivariable regression models without preventing meaningful estimation of their independent effects. Consequently, calendar period was not entered as a covariate in the adjusted analyses. This methodological constraint reflects the observational nature of the study and indicates that the observed differences should be interpreted as associations between care periods rather than definitive causal evidence of superiority of one supplementation strategy. The multicenter nature of the cohort further raises the possibility of center-level practice variation, including differences in counseling intensity, workflow organization, and follow-up implementation. However, center-stratified analyses demonstrated directionally consistent associations between BSM use and early complete correction across all participating centers, with no reversal of effect observed. Although center-specific confidence intervals were wide due to limited subgroup sample sizes, these findings argue against a single-center-driven effect and support the robustness of the primary association. Our results are consistent with prior literature documenting the high prevalence and clinical relevance of MD in candidates for bariatric surgery [4,10]. Several studies have shown that deficiencies in vitamin D, iron, folate, vitamin B12, zinc, and selenium are highly prevalent before surgery [8,16]. These abnormalities are multifactorial, reflecting poor dietary quality, chronic low-grade inflammation, altered micronutrient metabolism, and adipose tissue sequestration, and they frequently coexist within the same individual [4,12,17]. The coexistence of multiple deficiencies represents a clinically meaningful condition that challenges supplementation strategies focused on correcting individual micronutrients in isolation. Preoperative MD have important clinical implications. Inadequate nutritional status before surgery has been associated with anemia, impaired wound healing, fatigue, neurological manifestations, and delayed postoperative recovery [4,8]. Moreover, bariatric procedures may exacerbate pre-existing deficiencies or precipitate new ones through reduced gastric acid secretion, altered gastrointestinal anatomy, and decreased absorptive capacity [11,18]. Previous clinical observations suggest that patients who undergo systematic correction of deficiencies before surgery are less likely to develop early postoperative deficiencies, even when standardized postoperative supplementation is prescribed [9,19]. Despite guideline recommendations, preoperative supplementation practices remain heterogeneous [4,10]. Conventional approaches rely on targeted supplementation protocols that often require multiple products, potentially increasing regimen complexity and negatively affecting adherence, particularly in bariatric populations, where long-term compliance is already challenging [20,21,22,23]. Regimen complexity therefore represents a plausible barrier to effective real-world correction of MD. In the present study, the use of a BSM was associated with more than a twofold increase in the likelihood of achieving complete multi-deficiency correction at 4 weeks compared with CTS. Notably, several micronutrients in the CTS group were prescribed at equal or higher individual daily doses compared with those provided in the BSM formulation. However, differences in overall micronutrient composition and cumulative dosing between the two strategies may still have contributed to the observed effects. Because the present study was not designed as a controlled dose-comparison trial, the relative contribution of regimen simplicity versus differences in total micronutrient dosing cannot be fully disentangled. Treatment adherence emerged as an important interpretative component of these findings. Reduced supplementation burden is a recognized driver of improved compliance across chronic conditions, and a higher proportion of patients in the BSM group reported full adherence at 4 weeks compared with those receiving CTS. However, adherence was assessed through self-reported information documented in routine clinical records rather than through validated adherence questionnaires, pill counts, or pharmacy refill data. These reports were obtained during routine follow-up consultations with clinicians or dietitians and recorded in the clinical documentation as part of the standard preoperative bariatric evaluation. Self-reported adherence is known to overestimate true compliance and may be influenced by recall or social desirability bias. Although adherence assessment procedures were consistent across centers, differences in patient reporting behavior across calendar periods cannot be entirely excluded. Consequently, while higher adherence likely contributed to the increased probability of early correction observed in the BSM group, the present data do not allow definitive attribution of biochemical differences to adherence alone. The observed differences in “time to correction” reflect biochemical reassessment at predefined follow-up visits (4 and 8 weeks) rather than continuous time-to-event monitoring. Thus, the outcome should be interpreted as an operational measure of correction efficiency within routine clinical practice rather than as a precise estimate of biological normalization kinetics. Several limitations warrant consideration. The retrospective observational design and non-concurrent cohorts preclude causal inference and may be subject to residual confounding and temporal bias. Adherence was assessed using non-validated self-report measures, which may overestimate true compliance. In addition, several clinically relevant outcomes were beyond the scope of the present analysis. Postoperative micronutrient status, surgical outcomes, time-to-surgery intervals, and formal cost-effectiveness analyses comparing simplified versus targeted supplementation strategies were not systematically captured in the study dataset. Future prospective studies incorporating formal sample size estimation will be important to confirm the present findings. Furthermore, although the micronutrient composition of the bariatric-specific multivitamin differed slightly from individualized targeted regimens, sometimes providing lower doses of specific nutrients, the present study was not designed as a controlled head-to-head dose comparison. Despite these limitations, the study has important strengths, including its multicenter design, large sample size, strict inclusion criteria requiring multiple concurrent deficiencies, standardized laboratory assessments, and real-world clinical setting, all of which enhance the robustness and applicability of the findings. Overall, the present results should be interpreted as hypothesis-generating and support the feasibility and potential advantages of simplified preoperative supplementation strategies. Future prospective studies incorporating concurrent comparison groups, standardized dosing comparisons, validated adherence measures, postoperative nutritional outcomes, surgical pathway metrics, and economic evaluations will be important to better define the clinical and organizational impact of simplified micronutrient correction strategies.

## 5. Conclusions

In this multicenter real-world cohort of bariatric surgery candidates with multiple MD, the use of a bariatric-specific multivitamin was associated with a higher probability of achieving complete biochemical correction at 4 weeks compared with conventional targeted supplementation. The simplified regimen was also associated with a lower supplementation burden and higher self-reported adherence within routine clinical practice.

These findings suggest that simplified supplementation strategies may represent a practical approach to facilitate early preoperative nutritional optimization in bariatric surgery candidates presenting with multiple concurrent deficiencies. However, given the retrospective observational design and the non-concurrent comparison periods, the results should be interpreted as associative rather than causal.

Prospective studies with concurrent comparison groups, standardized dosing strategies, objective adherence assessment, and evaluation of postoperative nutritional and clinical outcomes are warranted to confirm these findings and to better define the clinical and organizational implications of simplified preoperative micronutrient correction strategies.

## Figures and Tables

**Table 1:** Baseline characteristics of the overall study population (*n* = 1560).

Variable	CTS (*n* = 620)	BSM (*n* = 940)
**Age (years), mean ± SD**	45 ± 13	43 ± 6
**Female sex, *n* (%)**	415 (67%)	658 (70%)
**BMI (kg/m^2^), mean ± SD**	52.3 ± 13.9	54.6 ± 11.4
**Vitamin D deficiency, *n* (%)**	403 (65%)	658 (70%)
**Iron deficiency, *n* (%)**	279 (45%)	470 (50%)
**Folate deficiency, *n* (%)**	186 (30%)	273 (29%)
**Vitamin B12 deficiency, *n* (%)**	155 (25%)	244 (26%)
**Zinc deficiency, *n* (%)**	124 (20%)	188 (20%)

SD: standard deviation; CTS: conventional targeted supplementation; BSM: bariatric-specific multivitamins.

**Table 2:** Baseline characteristics of the analytic cohort (patients with ≥3 baseline deficiencies; *n* = 216).

Variable	CTS (*n* = 82)	BSM (*n* = 134)
**Age (years), mean ± SD**	44 ± 11	42 ± 7
**Female sex, *n* (%)**	55 (67%)	93 (69%)
**BMI (kg/m^2^), mean ± SD**	53.1 ± 12.8	55.0 ± 10.9
**Mean number of baseline deficiencies**	3.4 ± 0.6	3.5 ± 0.7
**Vitamin D deficiency, *n* (%)**	71 (87%)	120 (90%)
**Iron deficiency, *n* (%)**	59 (72%)	100 (75%)
**Folate deficiency, *n* (%)**	48 (59%)	82 (61%)
**Vitamin B12 deficiency, *n* (%)**	44 (54%)	70 (52%)
**Zinc deficiency, *n* (%)**	36 (44%)	63 (47%)

SD: standard deviation; CTS: conventional targeted supplementation; BSM: bariatric-specific multivitamins.

**Table 3:** Primary and sensitivity analyses for complete correction of all baseline MD at 4 weeks (analytic cohort, *n* = 216).

Analysis	CTS (*n* = 82)	BSM (*n* = 134)	Absolute Risk Difference (95% CI)	Risk Ratio (95% CI)
**Primary analysis (overall analytic cohort)**	13/82 (15.9%)	55/134 (41.0%)	25.2 pp (7.8 to 40.0)	2.59 (1.51 to 4.44)
**Restricted follow-up window (28 ± 7 days)**	13/82 (15.9%)	53/132 (40.2%)	24.3 pp (6.9 to 39.2)	2.53 (1.48 to 4.35)
**Excluding early baseline months (January–March in available calendar)**	10/68 (14.7%)	39/93 (41.9%)	27.2 pp (7.4 to 43.9)	2.85 (1.53 to 5.30)
**Adjusted model (center fixed effects + baseline deficiency burden)**	15.4% (adjusted probability)	41.6% (adjusted probability)	26.3 pp (14.4 to 38.2)	2.69 (1.58 to 4.57)

CTS: conventional targeted supplementation; BSM: bariatric-specific multivitamins.

**Table 4:** Supplementation burden and treatment adherence in the analytic cohort (*n* = 216).

Outcome	CTS (*n* = 82)	BSM (*n* = 134)
**Mean number of daily supplement products**	3.5 ± 1.2	1.0 ± 0.0
**Full adherence at 4 weeks, *n* (%)**	57 (70%)	123 (92%)

CTS: conventional targeted supplementation; BSM: bariatric-specific multivitamins.

## Data Availability

The data included in this manuscript derived from the University database. We are not authorized to share the data with third party organizations. However, the corresponding author is available to provide any explanation to the Editor if requested.

## Supplementary Materials

The following supporting information can be downloaded at: https://www.mdpi.com/article/10.3390/nu18071047/s1, Figure S1: Forest plot of center-specific risk ratios (RRs) for early complete correction at 4 weeks (BSM vs. CTS) among patients with ≥3 baseline deficiencies; Table S1: Center-specific early complete correction of all baseline micronutrient deficiencies at 4 weeks among patients presenting with ≥3 baseline deficiencies (BSM vs. CTS).
