# Supplementary material for: Effectiveness of a Bariatric-Specific Multivitamin Versus Conventional Targeted Supplementation for Preoperative Micronutrient Deficiency Correction in Bariatric Surgery Candidates: A Multicenter Retrospective Cohort Study
Source: Nutrients. 2026 Mar 25;18(7):1047. doi: 10.3390/nu18071047 (PMC13074251; doi:10.3390/nu18071047)
Supplement: Supplementary file 1 [file nutrients-18-01047-s001.zip › Supplementary/Supplementary_Materials_S1_Figure.docx]

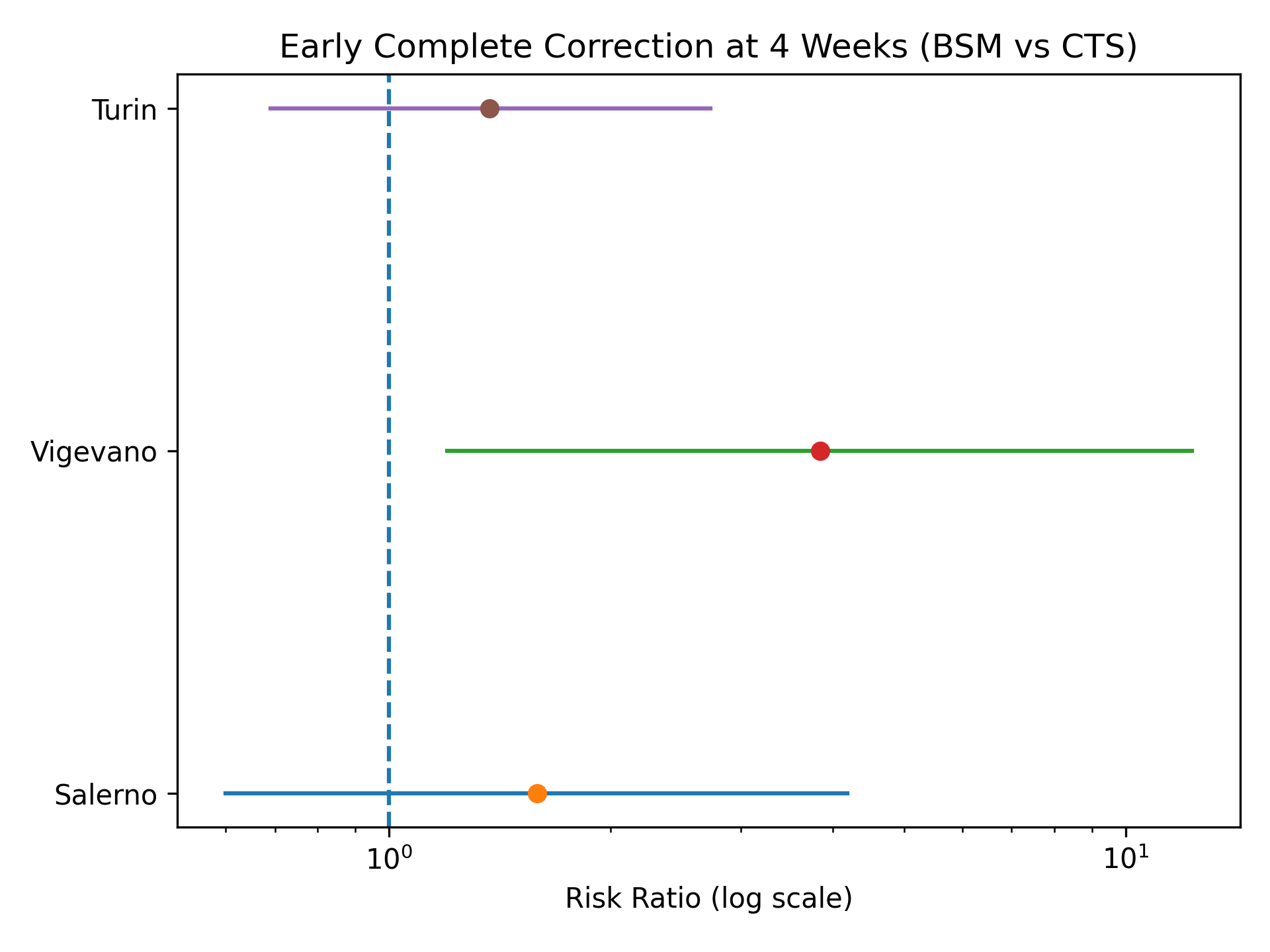


## Supplementary Figure S1. Forest plot of center-specific risk ratios (RRs) for early complete correction at 4 weeks (BSM vs CTS) among patients with ≥3 baseline deficiencies. Points indicate RRs and horizontal lines indicate 95% confidence intervals; the vertical dashed line represents RR = 1.
