# Supplementary material for: Effectiveness of a Bariatric-Specific Multivitamin Versus Conventional Targeted Supplementation for Preoperative Micronutrient Deficiency Correction in Bariatric Surgery Candidates: A Multicenter Retrospective Cohort Study
Source: Nutrients. 2026 Mar 25;18(7):1047. doi: 10.3390/nu18071047 (PMC13074251; doi:10.3390/nu18071047)
Supplement: Supplementary file 1 [file nutrients-18-01047-s001.zip › Supplementary/Supplementary_Materials_S1_Table.docx]

## Supplementary Table S1. Center-specific early complete correction of all baseline micronutrient deficiencies at 4 weeks among patients presenting with ≥3 baseline deficiencies (BSM vs CTS).

| **Center** | **CTS n/N (%)** | **BSM n/N (%)** | **Risk Ratio (95% CI)** |
| --- | --- | --- | --- |
| **Salerno** | 4/22 (18.2%) | 17/59 (28.8%) | 1.59 (0.60–4.19) |
| **Vigevano** | 3/32 (9.4%) | 13/36 (36.1%) | 3.85 (1.20–12.30) |
| **Turin** | 8/28 (28.6%) | 18/46 (39.1%) | 1.37 (0.69–2.73) |

CTS: conventional targeted supplementation; BSM: bariatric-specific multivitamins
